# Using metagenomic analyses to estimate the consequences of enrichment bias for pathogen detection

**DOI:** 10.1186/1756-0500-5-378

**Published:** 2012-07-27

**Authors:** James B Pettengill, Eugene McAvoy, James R White, Marc Allard, Eric Brown, Andrea Ottesen

**Affiliations:** 1 FDA Center for Food Safety and Applied Nutrition, Division of Microbiology, Molecular Methods and Subtyping, 5100 Paint Branch Parkway, College Park, MD, 20740, USA; 2 University of Florida - IFAS Hendry County Extension, PO Box 68, LaBelle, FL, 33975, USA; 3 IGS Institute for Genome Sciences, University of Maryland School of Medicine, 801 West Baltimore St, Baltimore, MD, 21201, USA

**Keywords:** Enrichment bias, Metagenomics, Pathogen, Taxonomy

## Abstract

**Background:**

Enriching environmental samples to increase the probability of detection has been standard practice throughout the history of microbiology. However, by its very nature, the process of enrichment creates a biased sample that may have unintended consequences for surveillance or resolving a pathogenic outbreak. With the advent of next-generation sequencing and metagenomic approaches, the possibility now exists to quantify enrichment bias at an unprecedented taxonomic breadth.

**Findings:**

We investigated differences in taxonomic profiles of three enriched and unenriched tomato phyllosphere samples taken from three different tomato fields (n = 18). 16S rRNA gene meteganomes were created for each of the 18 samples using 454/Roche’s pyrosequencing platform, resulting in a total of 165,259 sequences. Significantly different taxonomic profiles and abundances at a number of taxonomic levels were observed between the two treatments. Although as many as 28 putative *Salmonella* sequences were detected in enriched samples, there was no significant difference in the abundance of *Salmonella* between enriched and unenriched treatments.

**Conclusions:**

Our results illustrate that the process of enriching greatly alters the taxonomic profile of an environmental sample beyond that of the target organism. We also found evidence suggesting that enrichment may not increase the probability of detecting a target. In conclusion, our results further emphasize the need to develop metagenomics as a validated culture independent method for pathogen detection.

## Findings

Enrichment procedures are often used to increase the probability of detection of a particular pathogen. However, due to numerous factors including competition, differences in relative growth rates, growth inhibitors, and presence of bacteriophages, [1] enrichment results in a biased sample [i.e., enrichment bias; [2]. Given the objective of enriching, this bias is expected. However, our limited understanding of non-target effects that occur through the enrichment process make it difficult to rely on specific enrichment methods as best diagnostic approaches. Enrichment may have inherent and currently poorly understood consequences on resident microflora of a particular food or environmental sample, which may prove detrimental to the resolution of public health disease outbreaks.

Historically, quantifying enrichment bias was accomplished by determining the differences in relative abundance of a target organism (e.g., *Salmonella* spp., *Shigella* spp., *Listeria* ssp., or *Escherichia* spp.) among different enrichment treatments [1,3,4]. With the advent of next-generation sequencing and metagenomic methods, we can now describe the differences in incidence and potential abundance beyond the target organisms to that of nearly all bacterial lineages within a sample; our ability to do so will continue to improve with the increasing sequencing depth provided by next-generation sequencing methods and continually expanding reference databases. Metagenomic approaches also provide insight to ecological and functional dynamics associated with environments that host human pathogens, which in turn may increase our predictive ability to identify where a specific pathogen may arise. Although metagenomics has been used extensively to describe microbial communities, its utility for quantifying enrichment bias for public safety investigative purposes has yet to be fully explored [5].

In this study, we employed next-generation sequencing and a 16S rRNA metagenomic approach to evaluate the ways in which enrichment changes the taxonomic profile of a sample. We also investigate the effects of such a practice on the ability to detect the specific organism targeted by the enrichment procedure (i.e., the first step in the Bacterial Analysis Management (BAM) protocol for detection of *Salmonella* employed by the United States Food and Drug Administration (USFDA)). Differences in taxonomic profiles were characterized among 18 samples comprising 3 replicates each of enriched through universal pre-enrichment broth (UPB) and non-enriched tomato phyllosphere samples from three different sites surrounding Immokalee, Florida, USA, which is an area to which outbreak causing strains of *Salmonella* have been traced. We acknowledge that other culture independent methods exist for pathogen detection [e.g., quantitative PCR; [6,7], however, they are not well suited to quantifying enrichment bias and are not evaluated here.

**Figure 1:**
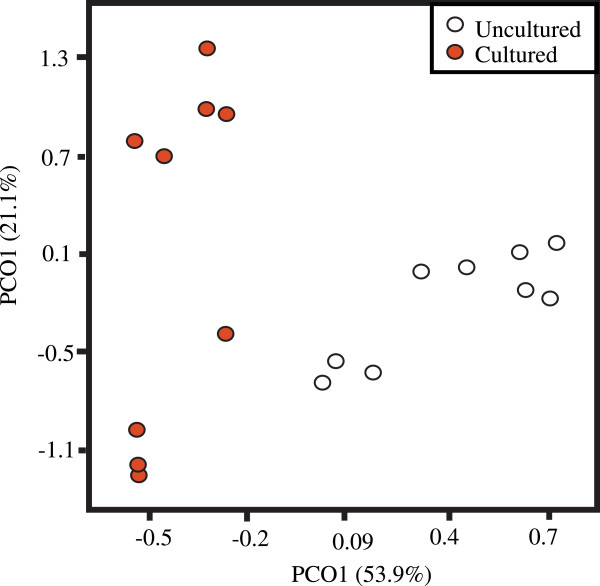
Principal coordinates analysis of the 18 samples comprising 3 replicates from 3 different locations for each the enriched and unenriched treatments.

From the analysis of 165,259 sequences (average number per sample = 9486; Table 1), we found based on a principal coordinates analysis (PCoA) that enriched and unenriched samples have different taxonomic profiles (Figure 1). At the Domain level, there was a statistically significant difference between the treatments in the abundance of Eukaryotes (*P* < 0.024) but not Bacteria (*P* = 0.367). This result is consistent with our results comparing Chao’s diversity index between the two treatments, which were not significantly different from one another (*P* = 0.12; Table 1).

**Table 1:** **Sampling locations, the number of cpDNA sequences, the number of sequences, Chao’s index, and two estimates of the number of*Salmonella*sequences for each of the 18 replicates**

**Site**	**Location**	**Treatment**	**Replicate**	**MG-RAST ID**	**cpDNA Sequences**	**Sequences**	**Chao’s Index**	***Salmonella*(Normalized)**	***Salmonella*(Raw)**
Florida47	26 27′ 42″ N	Enriched	1	4478869.3	0	10323	182.2	0	0
			2	4478871.3	0	8576	777.1	0	0
			3	4478873.3	3	9528	950.0	0	0
	081 26′ 16″ W	Unenriched	1	4478870.3	38	5852	493.2	0	0
			2	4478872.3	74	8965	414.7	0	0
			3	4478874.3	28	12235	593.7	0	0
BHN836	26 22′ 05″ N	Enriched	1	4478875.3	0	9972	1020.5	0	0
			2	4478877.3	1	9272	749.9	0	0
			3	4478879.3	1	12836	660.2	0	0
	081 15′ 59″ W	Unenriched	1	4478876.3	78	8786	848.4	0	0
			2	4478878.3	301	10804	1058.8	0	0
			3	4478880.3	195	8751	816.8	0	0
Soraya	26 17′ 12″ N	Enriched	1	4478881.3	2	7850	589.6	0.50	28
			2	4478883.3	0	7901	166.1	0.10	1
			3	4478885.3	1	7358	156.0	0	0
	081 20′ 21 W	Unenriched	1	4478882.3	1554	11911	1186.7	0	0
			2	4478884.3	2556	10382	1040.3	0	0
			3	4478886.3	669	9458	915.7	0	0
Total					5501	170760	701.11*	0.03*	1.61*

* Averages rather than sums.

**Figure 2:**
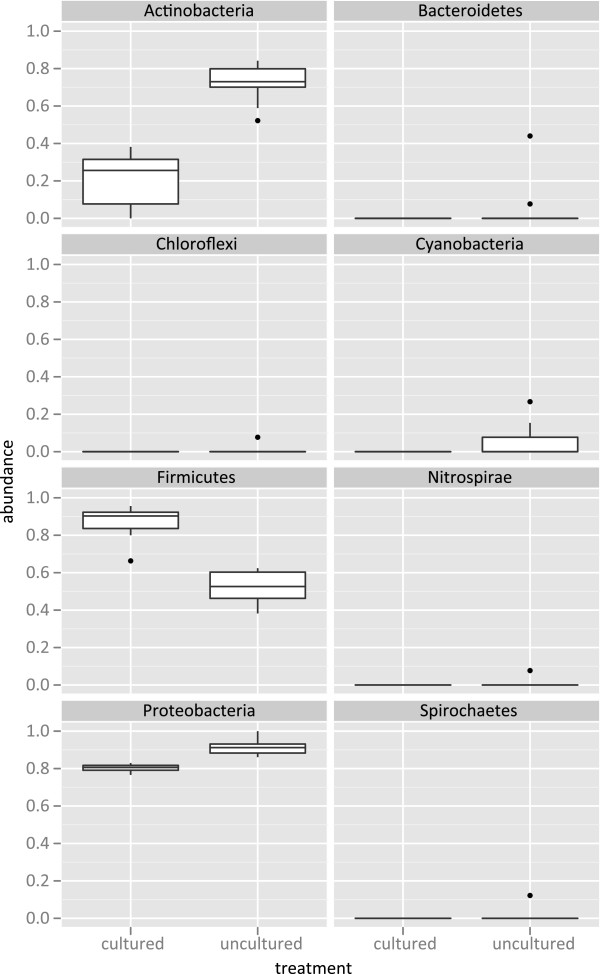
Boxplots illustrating the differences in normalized abundances of sequences assigned at the taxonomic level of bacterial phyla between the enriched and unenriched treatments.

The treatments differed significantly in the abundance of representatives of three different Bacterial phyla: Firmicutes were greater in the cultured samples (*t* = 8.28, *df* = 15.90, *p* < 0.001); Actinobacteria were greater in the uncultured samples (*t* = -8.33, *df* = 14.66, *p* < 0.001); and Proteobacteria were greater in the uncultured samples (*t* = -6.57, *df* = 11.64, *p* < 0.001) (Figure 2). The asymmetry in the abundance of taxa within those three phyla and lack of statistically significant differences among the other four bacterial phyla investigated (Bacteroidetes, Spirochaetes, Chloroflexi, and Nitrospirae) likely explains the insignificance of the differences between the treatments at the domain level for Bacteria.

Focusing on the Enterobacteriaceae, which includes *Salmonella*, we found evidence for significant differences (*p* < 0.05) in the abundance of five genera between the cultured and uncultured treatments (*Enterobacter, Klebsiella, Escherichia, Citrobacter,* and *Cronobacter*).

**Figure 3:**
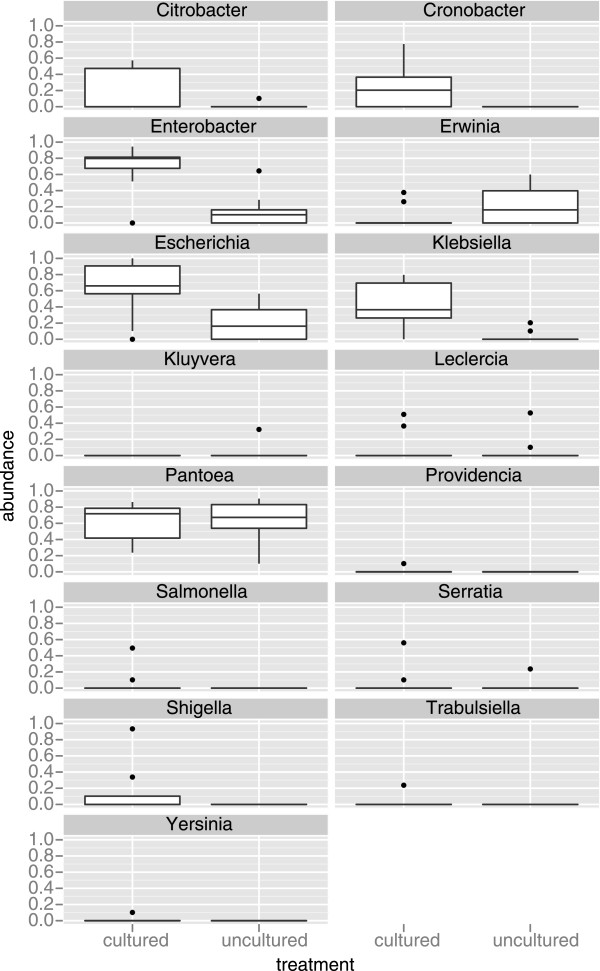
Boxplots illustrating the differences in normalized abundances of sequences assigned at the taxonomic level of genus within the family Bacteriaceae between the enriched and unenriched treatments.

Out of a total of approximately 54,000 sequences within the enriched samples we only found 28 sequences that were classified as *Salmonella* based on a best hit classification method. Given that only two of nine enriched samples contained putative *Salmonella* sequences, there was no significant difference between the two treatments in the abundance of *Salmonella* (*p* = 0.260; Table 1, Figure 3). We also found through further examination of the taxonomy of those sequences that they were just as likely to represent other species (e.g., *Panteoa* and/or *Klebsiella*). No sequences were assigned to *Salmonella* based on the lowest common ancestor approach. The results from the naïve Bayes classifier also provided evidence that cultured samples contained putative *Salmonella* sequences. However, we found that based on BLAST results of those sequences that they were also assigned to other taxa besides *Salmonella* and, therefore, we cannot say with certainty that they represent any one taxon. There were no putative *Salmonella* species found in the uncultured replicates regardless of classification method.

## Conclusions

Our results, which are among the first quantifying enrichment bias using a metagenomic approach, illustrate that the procedure of enriching a sample results in a drastically different taxonomic profile beyond that of the abundance of the target organism. This may not be of concern when there is certainty regarding the cause of an outbreak but if the organism responsible is unknown [so called ‘orphan’ microbes associated with diseases of previously unknown cause; [8] then our results suggest that enriching could greatly hinder the ability to identify those involved. For example, our results illustrate that enrichment using UPB significantly decreased the number of Actinobacteria, which is a taxonomic group that contains a number of human pathogens (e.g., *Tropheryma whipplei*). If a member of that group were responsible for an outbreak then our results suggest that the use of UPB may confound our ability to identify the causative agent.

**Figure 4:**
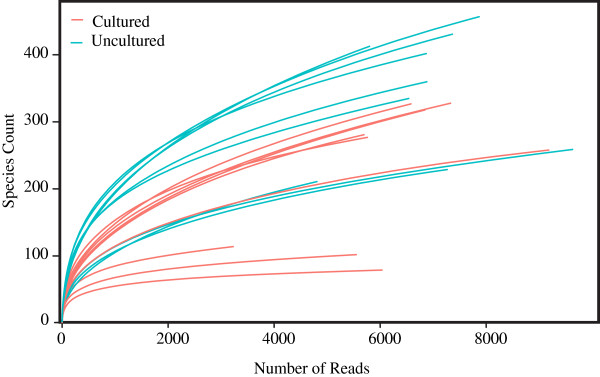
Rarefaction plots depicting the number of species detected as a function of the number sequence reads.

Second, we found that there was no significant difference between cultured and uncultured samples in the putative abundance of the organism, *Salmonella*, targeted by the first step in the *Salmonella* enrichment methods recommended by the FDA. The work of Jacobson et al. [9] that found that after following the BAM protocol only two-thirds of 540 samples artificially contaminated with *Salmonella* were positive for *Salmonella* further suggests that enrichment may not always result in detecting the target organism. Although it could be argued that with increased sequencing depth we would have detected a significant difference (Figure 4), a greater number of sequence reads could also have resulted in the detection of greater numbers of *Salmonella* within each treatment. Such a result would further support the results of this study. We expect that future studies will find the latter to be true. Therefore, as sequencing methods are developed that produce an even greater number of reads, metagenomics will likely become a validated tool for pathogen detection decreasing the need for enrichment.

## Methods

### Sampling and enrichment

Tomato samples were collected in May of 2011 from three different locations surrounding Immokalee, Florida, USA (Table 1). All samples were kept separate and brought back to the laboratory for processing. Below we briefly describe the enrichment protocol. For more detailed instructions see http://www.fda.gov/Food/ScienceResearch/LaboratoryMethods/BacteriologicalAnalyticalManualBAM/ucm070149.htm#Isol. Universal Pre-enrichment Broth (UPB) was added to samples of tomatoes at approximately 1.0 times the weight of the tomatoes, which was then incubated for 60 min at room temperature before being incubated at 35°C for 24 h.

### DNA extraction and PCR amplification

DNA from uncultured samples was extracted from a wash of tomatoes and leaves. The resulting wash was sonicated for 5 min before centrifugation to generate a pellet from which DNA was extracted. DNA from cultured samples was extracted from approximately 1 ml of overnight culture that was also spun down to create a pellet. Total DNA was extracted using the Promega Wizard DNA Purification Kit according to the manufacturer’s specifications.

16S fragments (V1–V3) were amplified for Roche pyrosequencing (454) using Roche Fusion Primer A, key, and MIDs (Multiplex identifiers) 27 through 44, and 27F: 5′ CGT ATC GCC TCC CTC GCG CCA TCAG (10 base pair MID) AGA GTT TGA TCC TGG CTC AG 3′ and Roche Fusion Primer B, key, no mid and 533R: 5′ CTA TGC GCC TTG CCA GCC CGC TCAG TTA CCG CGG CTG CTG GCA C 3′. Removal of PCR amplicons under 300 bases was performed using AmpPure XP from Agencourt at a ratio of 60 μl of AMPure beads to 100 μl PCR product. We used the above primers because 1) they created amplicons of suitable length for sequencing on the 454 machine, 2) have been used in previous studies detecting *Salmonella* [e.g., [10], and 3) have been validated in our lab where they were used to successfully amplify pure cultures of *Salmonella* ssp. *Newport*.

It is important to acknowledge that our results are based on analysis of 16S ribosomal DNA sequences obtained via PCR dependent methods, which can be considered an enrichment process that introduces its own potential biases. However, we have assumed that whatever bias may have been introduced through targeted sequencing was equal between the treatments and, thus, did not affect our conclusions regarding the effects on taxonomic profiles between the enriched and unenriched treatments. We also acknowledge that extraction procedures may represent another source of bias that can affect the taxonomic profile of a sample [e.g., [11]. Additional studies are necessary to determine whether extraction bias would affect the two treatments, enriched and unenriched, differently.

### Emulsion PCR and sequencing

Amplicons were diluted to 10^7^ molecules per μl and pooled to generate a mixture containing an equimolar representation of each independent replicate for subsequent emulsion PCR. Emulsion PCR was done using the Roche Lib-A MV kit according to the manufacturer’s specifications.

Approximately 800,000 enriched beads were loaded into one-quarter region of the Roche Titanium FLX pico-titer plate for sequencing on the Titanium FLX platform according to the manufacturer’s specifications. Sequencing read numbers were parsed in house with an adapted script to include MIDs beyond the 14 MIDs that Roche software automatically recognizes. Chimeric and chloroplast sequences were removed using the program ClovR [12]. Specifically, 12,913 were detected as being chimeric; 5,501 sequences were identified as being chloroplast DNA by the RDP classifier. Interestingly cultured samples had fewer sequences identified as chloroplast compared to uncultured replicates, however, this result was not significant (*t* = −2.0793, *df* = 8, *p* = 0.0712).

### Analyses

Sequences were uploaded into MG-RAST v3.1.2 [13], where they are also publicly available (Table 1). All analyses within MG-RAST were conducted using the following parameter settings: the RDP annotation source, maximum e-value = 1.0^−5^, minimum identity cutoff = 98%, minimum alignment length cutoff = 150 bp. We constructed rarefaction plots to estimate the limits of detection of our sequencing efforts (i.e., how well we were able to detect the taxonomic diversity within each sample).

As a first step to identify whether the cultured and uncultured replicates had different taxonomic profiles, we conducted a principal coordinates analysis (PCoA) on the normalized abundance counts of taxa within each replicate using the Bray-Curtis dissimilarity index. We also estimated Chao’s alpha diversity metric for each replicate using QIIME v1.4.0 [14]. Significance testing of the normalized abundances determined by MG-RAST and Chao’s diversity index were conducted grouping samples into two treatments (each with 9 replicates) and using Welch’s two-sample *t*-test as implemented in the stats package in R [15]. Using MG-RAST, we also identified the groups at different taxonomic levels that were responsible for the observed differences based on the PCoA. This was accomplished by comparing normalized abundances of a given taxonomic group between the different treatments with significance testing again done using a *t*-test.

Given our emphasis on the probability of detecting *Salmonella*, we determined the number of samples within each replicate that were identified as such using two different platforms. The first was MG-RAST within which the number of putative *Salmonella* sequences was determined based on the best-hit classification and lowest common ancestor approaches. The second platform we used was NBC (naïve Bayes classifier) that assigns sequences to species through a Bayesian framework with all bacterial genomes within NCBI serving as the reference database [16]. Because of the limited taxonomic breadth of the database used by NBC, we then used BLASTN [17] and the ‘nr’ database to further evaluate the taxonomic assignment of putative *Salmonella* sequences from the NBC analyses.

## Competing interests

The authors declare that they have no competing interests.

## Authors’ contributions

AO and EB conceived and designed the experiment. JBP and JRW conducted bioinformatic analyses. EM facilitated sample collection. MA provided the use of sequencing equipment. JBP and AO wrote the manuscript. All authors read and approved the final manuscript.
